# Individualized 3D-printed bolus promotes precise postmastectomy radiotherapy in patients receiving breast reconstruction

**DOI:** 10.3389/fonc.2023.1239636

**Published:** 2023-12-12

**Authors:** Jun Wang, Zhong-zheng Xiang, Chen-feng Tan, Yuan-yuan Zeng, Tian Yang, Xiao-yuan Wei, Si-ting Yu, Ze-lei Dai, Ning-yue Xu, Lei Liu

**Affiliations:** Division of Head & Neck Tumor Multimodality Treatment, Cancer Center, West, China Hospital, Sichuan University, Chengdu, Sichuan, China

**Keywords:** breast cancer, breast reconstruction, postmastectomy radiotherapy, 3d-printed bolus, dosimetry, skin toxicity

## Abstract

**Purpose:**

To evaluate the efficacy and safety of 3D-printed tissue compensations in breast cancer patients receiving breast reconstruction and postmastectomy radiotherapy (PMRT).

**Methods and materials:**

We enrolled patients with breast cancer receiving breast reconstruction and PMRT. The dose distribution of target and skin, conformability, and dose limit of organs at risk (OARs) were collected to evaluate the efficacy of the 3D-printed bolus. Radiation Therapy Oncology Group (RTOG) radiation injury classification was used to evaluated the skin toxicities.

**Results:**

A total of 30 patients diagnosed between October 2019 to July 2021 were included for analysis. Among all the patients, the 3D-printed bolus could ensure the dose coverage of planning target volume (PTV) [homogeneity index (HI) 0.12 (range: 0.08-0.18)], and the mean doses of D99%, D98%, D95%, D50%, D2% and Dmean were 4606.29cGy, 4797.04cGy, 4943.32cGy, 5216.07cGy, 5236.10cGy, 5440.28cGy and 5462.10cGy, respectively. The bolus demonstrated an excellent conformability, and the mean air gaps between the bolus and the chest wall in five quadrants were 0.04cm, 0.18cm, 0.04cm, 0.04cm and 0.07cm, respectively. In addition, the bolus had acceptable dosage limit of OARs [ipsilateral lung: Dmean 1198.68 cGy, V5 46.10%, V20 21.66%, V30 16.31%); heart: Dmean 395.40 cGy, V30 1.02%, V40 0.22%; spinal cord planning risk volume (PRV): Dmax 1634 cGy] and skin toxicity (grade 1, 76.0%; grade 2, 21.0%; grade 3, 3.3%).

**Conclusion:**

The 3D-printed bolus offers advantages in terms of dose uniformity and controllable skin toxicities in patients receiving breast reconstruction and PMRT. Further research is needed to comprehensively evaluate the effectiveness of the 3Dprinted bolus in this patient subset.

## Introduction

Breast cancer has become the most common malignancy in female, with 2.3 million new cases reported in 2020 (1, 2). Multidisciplinary treatment model, including surgery, chemotherapy, radiotherapy, endocrine therapy and anti-human epidermal growth factor receptor 2 (HER-2) based targeted therapy, is the standard of care for breast cancer (3). Breast reconstruction is increasingly acceptable due to its well cosmetic results and improved quality of life. The selection of reconstruction should take into account patient’s willingness, tissue availability and subsequent adjuvant treatments, especially for postmastectomy radiotherapy (PMRT) (4, 5).

The administration of PMRT in patients with breast cancer was depended on high risk factors, such as younger age, positive margin, advanced tumor stage, positive axillary lymph nodes, histological grade, and lymphatic vascular invasion, and previous study has demonstrated that PMRT can reduce the risk of locoregional recurrence (LR) and obtain survival benefits for these patients (6, 7). However, for patients receiving mastectomy, the normal structure of the breast has changed, and PMRT might cause insufficient dose to chest wall and increased dose to organs at risks (OARs) due to the dose build-up effect, leading to serious complications (skin necrosis, infection, pain, and impaired wound healing) and impaired quality of life (8–10). Therefore, it is important to overcome dose build-up effect and improve the dose of chest wall.

Tissue-equivalent bolus has been recommended routinely for patients receiving PMRT to improve dose uniformity and protect the OARs during radiation (11, 12). However, existing commercials bolus, such as silica, wax and thermoplastic material, cannot fit chest wall properly and create air gaps due to the complex anatomical contours of the chest wall after surgery, resulting in a loss of radiation dose at the lesion location (13, 14). The potential skin toxicities caused by the use of bolus outweigh its advantage of ensuring adequate dose, especially for patients receiving post-mastectomy reconstruction, which makes its application during radiation still a controversial task (15, 16). The 3D-printed bolus has emerged as a new technology that could optimize dose distribution and overcome the limitations of traditional commercial bolus by providing better conformance to the chest wall, and being more safe, environmental-friendly and durable (17). Nevertheless, there is little known regarding the use of bolus for patients with breast reconstruction during PMRT (18). Therefore, this study aims to evaluate the efficacy and safety of 3D-printed equivalent tissue compensations in patients receiving mastectomy and breast reconstruction, and PMRT, to ensure better precision radiation for breast cancer patients.

## Materials and methods

### Patient inclusion

This observational study enrolled patients who were treated at West China Hospital, Sichuan University from October 2019 to July 2021. The inclusion criteria were as follows: 1) pathologically confirmed with invasive breast cancer; 2) treated with mastectomy and post-mastectomy breast reconstruction; 3) patients with primary tumor > 5cm, or ≥ 4 positive axillary nodes, or 1-3 positive axillary nodes with multiple high- risk factors, such as younger age, poor-differentiated or undifferentiated, HER-2 positive disease, triple negative disease, and high ki-67 index; 4) having willing to receiving PMRT. Male patients, patients aged ≤ 18 years, and patients with distant metastasis at diagnosis were excluded. This study was approved by the Biomedical Ethics Committee of West China hospital, Sichuan University (Approval number: 2021-900). All included patients signed written informed consent at initial diagnosis.

### Fabrication of the 3D-printed bolus

The images of the chest contour were obtained using the computed tomography (CT) scan, and were stored in digital imaging and communications in medicine (DICOM) format, and then converted into a format namely the stereolithography file. The 3D- printing software (Mimics 10.01) was used to create an individualized bolus based on CT images of the patients. The present study employed silicone material to fabricate the individualized bolus. With a physical density similar to human chest wall skin, silicone efficiently mitigates the interference caused by dose build-up effects in PMRT, ensuring precise radiation. Compared to other commercial materials, silicone exhibits characteristics such as resistance to deformation, high flexibility, long-term bendability, and resilience to deterioration during extended use. Overall, the 3D-printed bolus utilized in this study proves to be cost-effective and environmentally friendly. The positioning fixator was used to fix the bolus to reduce the positioning error. The conformability of the 3D-printed bolus was daily verified by cone beam computed tomography (CBCT).

### The treatment planning and dosimetric evaluation

**Figure 1:**
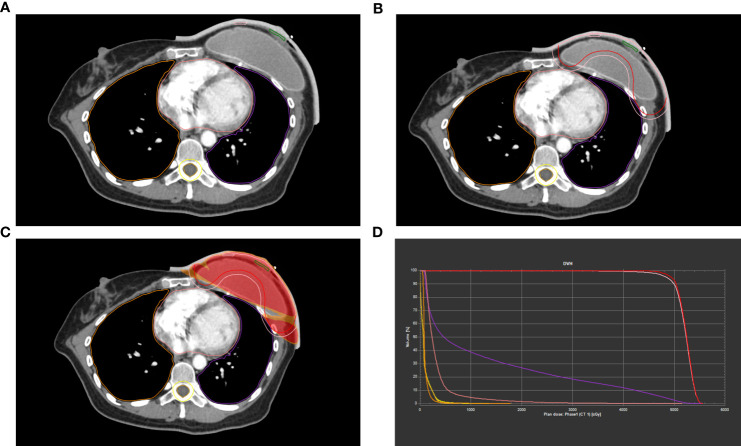
Example of dose distributions of patients with breast reconstruction using a 3D-printed bolus in TPS. **(A-C)** Contours of the target volume. **(D)** The Dose Volume Histogram (DVH) curve.

The treatment planning was designed in the Raystation treatment planning system (TPS) (version 4.7.5; Ray Search Laboratories AB, Stockholm, Sweden) (**Figure 1**). The clinical target volume (CTV) included the chest wall and supra and infraclavicular region with a planned dose of 50 gray/25 fractions, and the planning target volume (PTV) was defined as clinical target volume (CTV) with a certain 5mm margin. The PTV for the chest wall was denoted as PTV1, while the PTV for lymph drainage area was labeled as PTV2. In PTV2, the skin was cropped by 3mm from the body. As the chest wall was covered by the 3D-printed bolus, the skin was not cropped from the body in PTV1. The intensity modulated radiation therapy (IMRT) or volumetric modulated arc therapy (VMAT) radiotherapy techniques were used for radiation treatment planning. The dosimetric evaluation of PTV used the following parameters: D99%, D98%, D95%, Dmean, D50%, D2%, homogeneity index (HI = (D2%-D98%)/D50%), and absolute percentage differences (|%diff|=|100* (Dfact- Dtheory)/Dtheory|). The dose limit of OARs was evaluated as follows: ipsilateral lung (Dmean, V5, V20, V30), heart (Dmean, V30, V40), and spinal cord PRV Dmax) (19). Software Film QA Pro 2016 was used to analyze the dose distribution of the target volume and OARs.

### *In vivo* skin dose and conformability assessment

A 3D-printed bolus was applied to the entire chest wall for *in vivo* dosimetry measurements following radiation. GafChromic EBT3 (International Specialty Products, Wayne, NJ, USA) was used to measure the absorbed skin dose in radiotherapy (20). The method of skin dose assessment was described in detail in our previous study (21). In our study, skin dose was measured by creating a volume in the TPS. Eight GafChromic EBT3 films, each sized 3 x 2 cm², were placed between the 3D-printed bolus and the chest wall. Within each EBT3 film, we identified a central 1 x 1 cm² region of interest (ROI). Each ROI were delineated in the center of each sub-region, with a size of 1 × 0.1 cm, between the 3D-printed bolus and the patient’s skin across three-slice CT images. The conformability of the 3D-printed bolus was assessed by measuring the air gaps between bolus and chest wall in 5 quadrants: center, upper outer, upper inner, lower outer, lower inner, in chest CT images. The max, mix, and mean gap values in above-mentioned quadrants were used to assess the conformability of the 3D printed bolus.

### The evaluation of skin toxicity

Radiation Therapy Oncology Group (RTOG) radiation injury classification was used to evaluated the skin toxicities during and after PMRT (22). The grades of the skin toxicity were as follows: grade 1, mild erythema, dry desquamation or both; grade 2, moderate erythema or patchy moist desquamation; grade 3, confluent moist desquamation, pitting edema and tenderness; grade 4, necrosis, ulceration, or bleeding. The classification of the skin toxicity was assessed by 2 or 3 radiotherapists to guarantee the accuracy. All patients were visited once a week from initial radiotherapy to four weeks after PMRT. Patients were advised better to keep the irradiated chest wall dry, minimize the friction and use radioactive skin protectants during radiation. Not all patients utilized the 3D-printed bolus throughout radiotherapy. The decision to continue bolus usage was determined by assessing skin toxicities after 3-4 weeks of radiotherapy. Patients with grade 2 skin toxicities, thin chest wall skin, and a low risk of local recurrence, or those with grade 3 skin toxicities should discontinue bolus usage.

## Results

### Patient characteristics

A total of 360 patients diagnosed between October 2019 and July 2021 in our institution were enrolled. At finally, only 41 patients received breast reconstruction and PMRT, while 30 of them were included for analysis. The median age of the patients was 40 years old (range: 24-57 years). The left and right sites of the tumor were 43.9% (n=18) and 56.10% (n=23), respectively. The majority of the patients were T2-3 stage (75.7%, n=31), and N1-2 stage (75.6%, n=31). According to the 8th edition of the American Joint Committee on Cancer (AJCC) staging system of breast cancer, 9.8% (n=4), 31.7% (n=13), 39.0% (n=16), 4.9% (n=2), and 14.6% (n=6) of the patients had stage IIA, IIB, IIIA, IIIB, and IIIC diseases, respectively. The detailed information was showed in **Table 1**.

**Table 1:** Patient characteristics.

		n=41
Age (years)		
	Median (range)	40 (24-57)
Lesion sites		
	Left, n (%)	18 (43.9)
	Right, n (%)	23 (56.1)
T stage		
	T1, n (%)	7 (17.1)
	T2, n (%)	22 (53.7)
	T3, n (%)	9 (22.0)
	T4, n (%)	3 (7.3)
N stage		
	N0, n (%)	4 (9.8)
	N1, n (%)	19 (46.3)
	N2, n (%)	12 (29.3)
	N3, n (%)	6 (14.6)
Clinical stage		
	II A, n (%)	4 (9.8)
	II B, n (%)	13 (31.7)
	III A, n (%)	16 (39.0)
	III B, n (%)	2 (4.9)
	III C, n (%)	6 (14.6)

T-stage, clinical tumor stages; N-stage, clinical node stages; AJCC, American Joint Committee on Cancer (8th); ER, estrogen receptor negative; PR, progesterone receptor negative; HER2, human epidermal growth factor receptor 2.

### Dosimetry evaluation

The dosimetric characteristics of the target volume and OARs are presented in **Table 2**. Among all the patients, the mean doses of D99%, D98%, D95%, D50%, D2%, Dmax and Dmean of PTV were 4606.29cGy (4026-4871cGy), 4797.04cGy (4504-4949cGy), 4943.32cGy (4006-5055cGy), 5216.07cGy (5155-5271cGy), 5236.10cGy (5169-5302cGy), 5440.28cGy (5303-5563cGy) and 5462.10cGy (5320-5605cGy), respectively. The mean, max, and mix of HI values were 0.12, 0.18, and 0.08, respectively. With regard to the limit dose of OARs, the mean doses of Dmean of ipsilateral lung and heart were 1198.68cGy (range: 201-1634cGy) and 395.40cGy (range: 339-442cGy), respectively. The mean values of V5, V20 and V30 of the ipsilateral lung were 46.10%, 21.66% and 16.31%, respectively, and the mean values of V30 and V40 of the heart were 1.02% and 0.22%, respectively. The mean dose of Dmax of Spinal cord PRV was 1634cGy.

**Table 2:** The planning dose parameter.

Dose coverage		Mean	Max	Min
PTV	D_99%_ (cGy)	4606.29	4871	4026
	D_98%_ (cGy)	4797.04	4949	4504
	D_95%_ (cGy)	4943.32	5055	4006
	D_mean_ (cGy)	5216.07	5271	5155
	D_50%_ (cGy)	5236.10	5302	5169
	D_2%_ (cGy)	5440.28	5563	5303
	D_max_ (cGy)	5462.10	5605	5320
	D_mean_ (cGy)	5216.07	5271	5155
	HI	0.12	0.18	0.08
Spinal cord	D_max_ (cGy)	664.41	1570	72
Spinal cord PRV	D_max_ (cGy)	798.28	1634	78
Lung (ipsilateral)	V30 (%)	16.31%	19.84%	0.00%
	V20 (%)	21.66%	28.14%	0.00%
	V5 (%)	46.10%	54.44%	4.37%
	D_mean_ (cGy)	1198.68	1394.00	201.00
Heart	V30 (%)	1.02	3.01	0.07
	V40 (%)	0.22	0.89	0.00
	D_mean_ (cGy)	395.40	442.00	339.00

D99%, D98%, D95%, D50% and D2% is the dose of 99%, 98%, 95%, 50% and 2% PTV volume respectively. PTV, planning target volume. PRV, planning risk volume. V5, V20, V30 and V40 is the percentage volume receiving 5,20,30,40 Gy, respectively. HI, homogeneity index, HI = D2%-D98%/D50%.

### *In vivo* skin dose and conformability assessment

*In vivo* skin surface doses were measured in 4 of 30 patients. The mean values of Dmean were 210.69cGy (209.14-211.59cGy) and 209.97cGy (205.04-214.42cGy) in theory and measurement, respectively. The average of the absolute difference percentage was 0.33% (0.06-1.96), which mean that the actual dose closely matched the theoretical dose. The detailed information was showed in **Table 3**.

**Table 3:** Dose distribution in 7 points of the chest wall in 4 patients.

	P* 1	P 2	P 3	P 4	P 5	P 6	P 7	D_fact_	D_theory_	differ%|
	(cGy)	(cGy)	(cGy)	(cGy)	(cGy)	(cGy)	(cGy)	(cGy)	(cGy)	
P1	215.50	212.70	205.50	213.60	205.30	212.40	215.20	211.46	211.59	0.06
P2	195.50	203.40	202.95	204.70	204.45	210.20	214.10	205.04	209.14	1.96
P3	214.50	208.30	207.20	206.70	198.10	210.30	217.50	208.94	210.67	0.82
P4	215.30	214.60	211.95	215.10	214.60	210.20	219.20	214.42	211.36	1.53
Mean	210.20	209.75	206.90	210.03	205.61	210.78	216.50	209.97	210.69	0.33

*P, point; Dfact, fact radiation dose for chest wall skin; Dtheory, theoretical radiation dose for chest wall skin; |differ%|, |differ%|=|100*(Dfact- Dtheory)/Dtheory|, the absolute differences between theoretical and fact dose at the skin surface.

In addition, we evaluated the conformability of the 3D-printed bolus (**Table 4**). The mean air gaps between the bolus and the chest wall in center, upper outer, upper inner, lower outer, and lower inner quadrants were 0.04 cm (range: 0-0.33), 0.18 cm (range: 0-0.68), 0.04 cm (range: 0-0.36), 0.04 cm (range: 0-0.26), and 0.07 cm (range: 0-0.47), respectively, which showed good conformability.

**Table 4:** The air gaps between the bolus and the chest wall in five regions.

	Centre (cm)	Upper Outer (cm)	Upper Inner (cm)	Lower Outer (cm)	Lower Inner (cm)
Mean	0.04	0.18	0.04	0.04	0.07
Max	0.33	0.68	0.36	0.26	0.47
Min	0.00	0.00	0.00	0.00	0.00

### Skin toxicities

Among the 30 patients receiving breast reconstruction and PMRT, one patient lost follow-up. At finally, 29 patients were included for skin toxicities evaluation. During the radiation, 22 (76.0%) and 6 (21.0%) patients developed grade 1 and grade 2 skin toxicities. One patient experienced grade3dermal toxicities, resulting in an interruption of radiation. Furthermore, grade 1 (63.64%, n=14) and grade 2 (100%, n=6) skin toxicities were more likely to occur at fractions 22-25 during radiation. Within 4 weeks after PMRT, there were 11 (38.0%), 11 (38.0%), 5 (17.0%), and 2 (7.0%) patients suffering grade 1-4 skin toxicities (**Table 5**).

**Table 5:** Skin toxicity during and within 4 weeks of radiation.

	During radiation	Within 4 weeks after radiation
Grade 1 (%)	22 (76.0)	11 (38.0)
Grade 2 (%)	6 (21.0)	11 (38.0)
Grade 3 (%)	1 (3.0)	5 (17.0)
Grade 4 (%)	0 (0.0)	2 (7.0)

## Discussion

This study aims to explore the dosimetric characteristics, effectiveness and safety of the 3D-printed bolus in patients with breast reconstruction during radiation. We summarized that the use of the 3D-printed bolus ensures sufficient skin dose, uniform dosage in chest wall, excellent vital organ limits and acceptable skin toxicity in this patient subset. Therefore, our individualized 3D printed bolus had excellent practicality and safety, and provided a more precise radiotherapy strategy for patients receiving reconstruction and PMRT.

Air gaps can result in inadequate radiation dose and consequently affect the effectiveness of radiation (23). Butson et al. (24) have shown that 4mm air gaps might cause a 4% reduction in the dose of high-energy X-ray beams. The anatomy of the chest wall changed after reconstruction, and previous commercial boluses couldn’t fit it well (25). The 3D-printed bolus was made from CT images, which could better match the contour of the chest wall. Our study indicates that customized 3D-printed bolus reduced unnecessary air gaps to 0.04cm, 0.18cm, 0.04cm, 0.04cm, and 0.07cmin center, upper outer, upper inner, lower outer, and lower inner quadrants respectively. Therefore, this 3D-printed bolus is a promising technology for clinical promotion (26).

The 3D-printed bolus provided a better dose distribution. The mean values of D98%, D95% and HI of PTV were 4797.04cGy, 4943.32% and 0.12 respectively. To ensure sufficient dose to the target volume, the radiation dose to the OARs is higher due to increased distance and arc between the skin and chest wall caused by reconstruction (27, 28). The mean values of Dmean and V20 of the ipsilateral lung were 1198.68cGy and 21.66%, which were lower than the values of 2000cGy an 30% reported previously (29). Nisha et al. (30) confirmed that the average V20 of ipsilateral lung of patients with reconstruction was significantly reduced (25.3% vs 41.4%, P<.0001), and the average lung dose was also lower (13.0Gy vs 18.0Gy, P<.0001) compared with patients without reconstruction. Our study showed that the mean values of Dmean and V30 of heart were 395.40cGy and 1.02%, indicating better heart protection in our study. Additionally, the average values of Dmean of heart in patients with/without reconstruction were 395.40cGy and 465.92cGy, respectively (p=0.030), both of which were lower than 800cGy (31). This suggested that the 3D-printed bolus may be more suitable for reconstruction patients. We analyzed *in vivo* dosimetry in 4 patients, and the results showed that the |differ%| was 0.33 (0.06, 1.96). We used IMRT and VMAT to reduce the impact of respiratory motion and positioning errors during radiation, which was more beneficial for accurate radiotherapy (32).

In our study,22 (76.0%) patients experienced grade I skin toxicities, and6 (21.0%) of them experienced grade II radiodermatitis after using the 3D-printed bolus. Dahn et al. (18) reported that the rate of grade III skin toxicities ranged from 45% to 88% when bolus was used daily. In contrast, only one patient in our study had to interrupt treatment due to grade III skin toxicity. Our findings were consistent with Gong (33), who showed that none of patients undergoing radiation with Thermoplastic Elastomer (TPE) bolus experienced grade III or IV radiodermatitis. As the number of radiation sessions increased, the likelihood of developing radiodermatitis also increased. Most Grade I/II skin toxicities occurred at the end of radiotherapy (f22-f25), which was consistent with Anabela (34)’s findings. We indicated that the 3D-printed bolus was made of silicone material, which is safer and more eco-friendly than traditional materials (35). Furthermore, patient education also played an important role in reducing the risks of skin toxicities during radiation. We taught patients to reduce skin friction, keep the chest wall skin dry and use radiation skin protectants.

Nevertheless, there are several limitations in our study. First, it is a single-center clinical study with a limited sample size, which has inherent confounding factors in non-randomized studies. Therefore, the results of our study need to be further verified in multi-center clinical trials with large sample size. Secondly, radiation might lead to capsular contracture of breast prosthesis; however, we have not collected related data to evaluate the effect of radiation on the breast prosthesis. Finally, there was no extended follow-up to analyze the survival outcomes of the study population, and we will further explore it in our later research.

## Conclusion

In conclusion, the customized 3D-printed bolus offers advantages in terms of dose uniformity and controllable skin toxicities, making it a promising option for clinical promotion in patients receiving mastectomy, breast reconstruction and PMRT. Further research is needed to comprehensively evaluate the effectiveness of the 3D-printed bolus in this patient subset.

## Data availability statement

The raw data supporting the conclusions of this article will be made available by the authors, without undue reservation.

## Ethics statement

The studies involving humans were approved by Biomedical Ethics Committee of West China hospital, Sichuan University (Approval number: 2021-900). The studies were conducted in accordance with the local legislation and institutional requirements. The participants provided their written informed consent to participate in this study.

## Author contributions

JW, Z-ZX and C-FT drafted the manuscript. LL conceived of the study. Y-YZ, TY, X-YW, S-TY, N-YX, and Z-LD acquired and organized the datasets. JW and C-FT conducted the statistical analyses. JW, Z-ZX, C-FT and LL participated in the study design. All authors read and approved the final manuscript.
